# Advances in Liver Transplantation: where are we in the pursuit of transplantation tolerance?

**DOI:** 10.1002/eji.202048875

**Published:** 2021-09-04

**Authors:** Niloufar Safinia, Trishan Vaikunthanathan, Robert Ian Lechler, Alberto Sanchez‐Fueyo, Giovanna Lombardi

**Affiliations:** ^1^ Division of Transplantation Immunology & Mucosal Biology King's College London London UK; ^2^ Department of Inflammation Biology King's College London London UK

**Keywords:** immunosuppression withdrawal, immunotherapy, liver transplantation, regulatory T cells, tolerance

## Abstract

Liver transplantation is the ultimate treatment option for end‐stage liver disease. Breakthroughs in surgical practice and immunosuppression have seen considerable advancements in survival after transplantation. However, the intricate management of immunosuppressive regimens, balancing desired immunological quiescence while minimizing toxicity has proven challenging. Diminishing improvements in long‐term morbidity and mortality have been inextricably linked with the protracted use of these medications. As such, there is now enormous interest to devise protocols that will allow us to minimize or completely withdraw immunosuppressants after transplantation. Immunosuppression withdrawal trials have proved the reality of tolerance following liver transplantation, however, without intervention will only occur after several years at the risk of potential cumulative immunosuppression‐related morbidity. Focus has now been directed at accelerating this phenomenon through tolerance‐inducing strategies. In this regard, efforts have seen the use of regulatory cell immunotherapy. Here we focus particularly on regulatory T cells, discussing preclinical data that propagated several clinical trials of adoptive cell therapy in liver transplantation. Furthermore, we describe efforts to further optimize the specificity and survival of regulatory cell therapy guided by concurrent immunomonitoring studies and the development of novel technologies including chimeric antigen receptors and co‐administration of low‐dose IL‐2.

## Graphical abstract

Long‐term survival in liver transplantation remains suboptimal secondary to the use of toxic immunosuppressants. Here, we review tolerizing immunotherapy strategies centered around the use, optimization, and expansion of regulatory T cells.

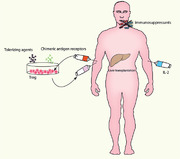

## Introduction

Liver transplantation is the definitive management for patients with end‐stage liver disease. Short‐term outcomes following transplantation have improved immensely in company with advances in surgical practices and immunosuppressive agents [1]. Long‐term outcomes, however, have drifted behind, marred by the very medications that gifted such favorable early survival rates following transplantation. It has become evident that the main causes of long‐term morbidity and mortality after transplantation are non‐hepatic and are either directly or indirectly linked to the requisite life‐long administration of non‐specific immunosuppression predisposing to cardiovascular disease, diabetes, infection, malignancy, and renal disease [2]. The current position of immunosuppressive medications in transplantation can no longer be considered acceptable. In support of this, there is now considerable interest in developing protocols to minimize, or better still, completely withdraw immunosuppression soon after transplantation to promote tolerance: the transplant panacea defined as a state of sustained immunological unresponsiveness to the transplant in the absence of chronic immunosuppression.

The liver is distinctively recognized as a tolerogenic organ translating to overall lower incidence and consequence of rejection, relative unimportance of human leukocyte antigen (HLA) incompatibility, and the permission of lower doses of immunosuppression in transplantation, making it the ideal candidate in which to develop and optimize tolerogenic protocols [3].

### Rationale for tolerance studies in the liver

**Figure 1:**
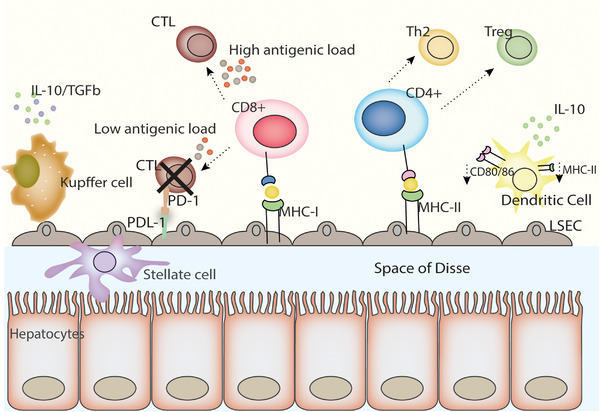
Tolerogenic properties of the liver: cellular interactions and antigen presentation within the liver. The liver has a tolerogenic microenvironment, functioning at a steady state to attenuate immune responses: Liver sinusoidal endothelial cells are covered in a porous epithelium, and form the interface of the hepatic sinusoid between blood and hepatocytes, separated by the space of Disse. LSECs present antigen to naïve CD8^+^ T cells, predisposing cytotoxic T Lymphocytes to apoptosis. LSEC antigen presentation to CD4^+^ T cells favors their differentiation to Treg and Th2 phenotypes. Hepatic stellate cells contribute to immunoregulation via PDL‐1‐ PD1 interaction and induction of apoptosis in cytotoxic T lymphocytes. Kupffer cells and liver resident macrophages produce immunoregulatory cytokines IL‐10 and TGF‐β promoting a tolerogenic environment. Liver dendritic cells have a tolerogenic phenotype, with lower expression of MHC class II and co‐stimulatory molecules and upon toll‐like receptor ligation secrete immunoregulatory cytokines IL‐10 and IL‐27. Abbreviations: CTL (cytotoxic T lymphocyte), Th2 (T‐helper 2), TLR, Toll‐like receptor, Treg (regulatory T cells), MHC (major histocompatibility complex), LSEC (liver sinusoidal endothelial cell)

The fundamental processes that gift the liver these tolerogenic properties in transplantation are not fully understood. However, various hypotheses have been put forward: the liver is positioned to receive 75% of its blood flow from the gastrointestinal tract, spleen, and its associated organs. While the intestinal mucosa acts as a primary barrier against microbial translocation it is by no means impenetrable. As such the liver is continually subject to antigen and endotoxin stimulation via the portal venous circulation. The liver is therefore designed to manage this antigen load through the presence of various specialized cells including, hepatocytes, Kuppfer cells, liver sinusoidal endothelial cells (LSEC), liver‐specific dendritic cells (DCs), and stellate cells. These cells form a specialized infrastructure within liver sinusoids allowing for intimate interactions with circulating lymphocytes supporting a delicate equilibrium between defensive immune responses against harmful antigens and immune tolerance [4]. Several of these cells act as effective antigen‐presenting cells (APC) for T lymphocytes; their interactions, however, are tailored towards tolerance through downregulation of co‐stimulatory molecules and preventing T cell activation, induction of apoptosis, release of immunoregulatory cytokines, and induction of regulatory T cells (Tregs) (**Figure** **1** **)**.

Another explanation surrounding the liver's unique tolerogenic qualities in transplantation is the induction of microchimerism by donor passenger leukocytes. Following transplantation, there is a migratory exchange of leucocytes between the graft and the recipient. Passenger leucocytes from the transplanted liver travel out into the recipient's blood stream and persist for a considerable amount of time thereafter developing a state of microchimerism [5]. These passenger leucocytes have been shown to traffic to lymphoid tissue precipitating a powerful immune response. Strong T cell activation leads to large quantities of proinflammatory cytokine production that paradoxically leads to the apoptosis of alloreactive T lymphocytes, and instead a state of hyporesponsiveness towards donor‐specific antigens [6].

A further rationalization relates to the size of the organ. The liver is the largest transplanted organ with significant tissue antigen mass which has been hypothesized to form a “cytokine sink” diluting cytokines and alloreactive T lymphocytes, in turn exhausting recipient immune responses. This is centered on the premise that a pivotal density of alloreactive T cells is required to cause rejection, and the significant antigen dose associated with liver transplantation thins the finite T cell repertoire thus not reaching this tipping point and in turn favoring tolerance [7].

Indeed, on account of these tolerogenic facets there are reports of patients post‐liver transplantation acquiring “operational” tolerance, permitting the withdrawal of immunosuppressive medication in the absence of graft rejection, highlighting the realistic potential for drug‐free transplantation [8, 9, 10].

### Spontaneous tolerance and immunosuppression withdrawal trials

In the early 1990s, anecdotal retrospective observations of spontaneous tolerance were noted in liver transplant recipients in cases of non‐compliance or immunosuppression cessation due to posttransplant lymphoproliferative disorders or infection [11, 12, 13, 14, 15]. Subsequently, various single‐center studies have ratified these observations, which have been the driving force behind recent and ongoing immunosuppression withdrawal trials. Cumulative evidence from these initial trials suggested operational tolerance existed in around 20% of liver transplant recipients [12, 16, 17, 18, 19, 20, 21, 22, 23], however this estimation does not acknowledge the variability and lack of standardization of the studied population making direct comparisons difficult. Several multicenter clinical trials have looked at immunosuppression withdrawal in pediatric and adult liver transplantation reporting states of operational tolerance ranging from 5.6% to 60% [9, 24‐27]. Benitez et al. highlighted a link with age and time from transplantation (NCT00647283). Here, liver transplant recipients achieved operational tolerance at a frequency of 13%, 38%, and 79% at <6 years, between 6 and 11 years, and >11 years post‐transplant, respectively [8].

More recently research has been directed on the choice of immunosuppressants as factors to determine successful immunosuppression withdrawal. Transplant immunosuppression protocols often consist of a cocktail of immunosuppressants including: induction agents, calcineurin inhibitors (CNI), and anti‐metabolites, and are mixed in the various regimens with respect to timing and dosing, with the selection of immunosuppressive agents, waxing and waning throughout the transplants lifespan, differing widely between centers. Recent data have suggested that the conversion from CNI‐based regimen, such as tacrolimus, to sirolimus, the mechanistic target of rapamycin inhibitor (mTOR‐I), enhances immunoregulatory profiles in liver transplant recipients through the enrichment of regulatory T cells [28]. As such, in their prospective clinical trial of sirolimus monotherapy withdrawal, Levitsky et al. investigated whether this strategy could foster tolerance induction in liver transplant recipients (NCT02062944). 15 patients were recruited, with non‐viral or non‐immune causes of liver disease and >3 years after liver transplantation, of which, eight (53%) achieved operational tolerance, six patients had biopsy‐proven mild acute rejection during immunosuppression weaning, and one patient was withdrawn from the study due to the recurrence of liver cancer. This study also proposed that pre‐weaning gene expression profiles and peripheral blood immunophenotyping may produce useful surrogate endpoints indicating successful mTOR‐I therapy withdrawal, further supporting the value of biomarker analysis to predict immune tolerance to inform immunosuppression withdrawal [23, 29, 30].

We must acknowledge the significant risk of rejection in trials of immunosuppression withdrawal and while spontaneous operational tolerance is a very realistic prospect, emphasis needs to be placed on prospective identification of these individuals prior to withdrawal. As such significant research has been conducted in identifying non‐invasive biomarkers to define immunological unresponsiveness indicative of operational tolerance and to also guide immunosuppression weaning through surrogate endpoints in tolerance induction trials (reviewed in [31]). With this in mind, multicenter immunosuppression withdrawal trials are incorporating biomarker analysis into their secondary endpoints. A study by Markmann et al. (NCT02533180, OPTIMAL) will aim to identify tolerance biomarkers following immunosuppression withdrawal, evaluating donor‐specific immune senescence and exhaustion. Additionally, we are conducting a prospective randomized clinical trial (NCT02498977, LIFT) with a comparable design, but concentrated on identifying biomarkers with transcriptional signatures unique to operationally tolerant individuals. Patients recruited into this study are post‐transplant at > 3 years if ≥50 years of age, or > 6 years if 18–49 years old, excluding patients with immune‐mediated liver disease and viral hepatitis.

It is worth mentioning that whilst these studies have demonstrated the seemingly encouraging frequency of spontaneous operational tolerance in the older population, several years after transplantation, it is still unclear whether cumulative immunosuppression exposure leading up to the point where withdrawal is considered may have already caused irreconcilable harm. This highlights the need for tolerance induction therapies to accelerate tolerance in those who arguably need it most, the young and recently transplanted.

Consequently, cellular therapies, utilizing tolerogenic immune cell populations have been put forward as a therapeutic strategy. Recent developments in cell manufacturing processes and technologies have highlighted the feasibility of their production with clinical trials providing the safety and efficacy data needed for their clinical application. The subsequent focus of this review will be on the role regulatory T cells (Tregs) have to play in accelerating tolerance induction in liver transplantation.

### Tregs in transplantation; Pre‐clinical data

Tregs form 5–10% of our total CD4^+^ T cell compartment [32] whose phenotype is delineated by high levels of the IL‐2 receptor α chain (IL‐2Rα, CD25^hi^) [33], low levels of CD127, and the intracellular transcription factor Forkhead box protein 3 (FOXP3), known to play a crucial role in their development and function [34]. Importantly, while FOXP3 expression cannot be ascribed as a sole defining feature of Tregs, with effector human T cells also transiently expressing FOXP3, the methylation status of a conserved region within the *FOXP3* gene (Treg specific demethylated region, TSDR) stands as a reliable indicator of Treg identity [35]. In line with this, *FOXP3* gene mutations have been associated with various autoimmune and hematological disorders [34], and Treg loss of FOXP3 has been shown to result in the acquisition of effector properties by these cells, with the production of pro‐inflammatory cytokines [36]. Moreover, research has suggested several mechanisms responsible for Treg suppressor function (reviewed in [37]).

Initial preclinical studies surrounding the role of Tregs in transplantation have been supported by mouse models of cardiac, islet, and skin transplantation demonstrating that the presence of Tregs are crucial in promoting and sustaining tolerance, further validated in Treg depletion studies [38, 39] (reviewed in [40]). Additionally, mouse models of bone marrow transplantation (BMT) [41, 42] and pancreatic islet cell transplantation [39] have not only reiterated the importance of Tregs in transplantation tolerance, but have also highlighted the role of adoptive Treg therapy, in either ameliorating graft‐versus‐host disease (GVHD) and facilitated engraftment or preventing graft rejection, respectively. Such experiments put forward the concept that tolerance‐inducing strategies require a priority of regulation through either increasing the number of *in vivo* Tregs over effector T cells by their adoptive transfer or by their *in vivo* modulation, namely by targeting Treg specific pathways in order to increase their number or suppressor function. In their study, Fan et al. elegantly demonstrated that the outcome of the transplanted allograft relies on the balance between effector and regulatory cells [43], supporting prior publications [44, 45] that the success of tolerance induction strategies will require a preconditioning regimen, including the reduction in the number of allospecific effector T cells. They further proposed that without such an approach, Tregs will be unable to control effector T cell responses and rejection will ensue.

While this review focuses and provides the rationale for the use of Tregs for tolerance induction, it is important to bear in mind the role of other immune cells and intrahepatic parenchymal cells during inflammation and liver allograft rejection. If Tregs are indeed to induce tolerance, then evidence needs to support their regulatory role on the various immune cells/ parenchymal cells involved in this process. In line with this Tregs have been shown to regulate a variety of immune cells of both myeloid and lymphoid lineages [46, 47], whose unopposed action can result in rejection (reviewed in [48]). Additionally, the phenomenon of “infectious tolerance” also describes the process whereby Tregs can induce a regulatory phenotype on other immune cells, a proposed mechanism of tolerance induction by these cells. In line with this we have previously shown the role of Tregs in inducing an alternative phenotype of monocytes, with a reduced ability to produce proinflammatory cytokines or to express MHC class II, with a reduced capacity to expand Th17 cells [49]. This effect was noted with the use of *in vitro* expanded Tregs as compared to freshly isolated Tregs, further supporting their use as a cell therapy product in view of the pre‐requisite of cell expansion prior their adoptive transfer [49]. In line with our findings, in the setting of viral‐induced myocarditis, the adoptive transfer of Tregs has been shown to dampen down the inflammatory response and prevent fibrosis, via induction of an anti‐inflammatory macrophage phenotype [50].

There are, however, still many questions to be answered and work from our laboratories and others has focused on enhancing the current Treg product for cell therapy application. Factors such as optimization of the Treg potency, persistence, and safety and the challenges faced with the manufacture of clinical‐grade Tregs have been reviewed in detail by our group [32, 49, 51].

#### Antigen specificity

The ideal Treg candidate for immunotherapy in the setting of transplantation is one with allospecificity, with the added advantage that the following injection they would be focused at the site of transplant, where they are required to exert their immunomodulatory function [52]. Furthermore, unwanted pan‐suppression, predisposing to off‐target consequences such as infection and malignancy, can be avoided.

In line with this, the past few years have seen publications, from us and others, generating Tregs specific to donor antigens showing these cells to be more effective at controlling graft rejection in preclinical models, as compared to nonspecific Tregs [53, 54]. Moreover, in later studies, Treg lines with direct allospecificity were generated by culture of human Tregs with APCs, such as B cells and dendritic cells, obtained from the donor, and their function was tested *in vivo* in humanized mouse models supporting their superiority over polyclonally expanded Tregs in the prevention of alloimmune mediated allograft damage [55, 56, 57]. Moreover, Zheng et al. proposed the use of mature B cells as compared to immature DCs to be more potent APCs to generate Tregs with superior suppressive capacity with higher expression of FOXP3 and CD25 expression [58]. It is important to note, however, that the studies published to date, using humanized mouse models transplanted with human cells or tissues, have thus far allowed the study of Tregs with direct allospecificity solely due to the nature of the animal models used. Here, immune‐deficient mice have been reconstituted with PBMC, resulting in the engraftment and activation of T cells with direct allospecificity [56, 57, 59, 60]. However, it is important to bear in mind that evidence suggests a role of the indirect pathway of allorecognition in both acute and chronic rejection and as such this is the pathway that would need to be regulated as well in order to achieve tolerance [53].

Moreover, we have recently provided further evidence in support of previously published work, reporting the additional advantage of combination therapy to increase the efficacy of allospecific Tregs [60, 61]. Using an MHC class I mismatched skin transplantation mouse model, we demonstrated the role of B cells in antigen presentation and activation of T cells with indirect allospecificity. We showed the depletion of B cells at the time of transplantation to result in the prolonged survival of the transplanted skin with further evidence highlighting the adoptive transfer of Tregs in a B cell deplete recipient to further prolong the survival of the graft.

Of note, research has also started to focus on gene transfer technology as a mechanism to generate antigen‐specific Tregs. In the field of transplantation, we generated Tregs with direct allospecificity by co‐culture with donor‐derived immature dendritic cell APCs and using T cell receptor (TCR) gene transduction we conferred additional indirect allospecificity to the same Tregs with direct specificity for MHC‐mismatched heart allografts. We have shown the effectiveness of these cells in inducing indefinite survival of the heart grafts, with Tregs with indirect pathway allospecificity required to prevent chronic vasculopathy [62]. In line with our findings, Brusko et al. showed the *in vivo* function of expanded human Tregs transduced with a TCR specific for a melanoma antigen tyrosinase in the context of HLA‐A*0201, using a tumor model. In their study they showed the adoptively transferred Tregs to inhibit effector T cells, including those with specificity for the tyrosinase, resulting in the increased tumor volume [63].

Additionally, Kim et al. transduced Tregs with a retroviral construct encoding for a TCR from a myelin‐basic protein (MBP) specific T‐cell clone of an MS patient [64]. They showed the activation of transduced Tregs *in vitro* in response to MBP peptide on HLA‐DR15 expressing APCs and using a human HLA DR15 transgenic mouse model, showed the *in vivo* function of these transduced Tregs in ameliorating immunopathology in a myelin oligodendrocyte glycoprotein (MOG)‐induced experimental allergic EAE mouse model.

There has been significant interest in the construction of antigen specificity through the use of chimeric antigen receptors (CAR) [65]. CARs are man‐made molecules manufactured to recognize specific proteins, redirecting the specificity of T cells. They are composed of an ectodomain, usually an scFv specific fragment recognizing the protein of interest, transmembrane domains, and an endodomain consisting of signaling molecules promoting cell activation. CARs were initially manufactured for targeted therapies in cancer treatment [66] and their subsequent clinical translation has been extremely successful [67, 68, 69, 70] (reviewed in [51]).

Further to their application in the setting of cancer and autoimmune diseases, CAR technologies have been translated to generate antigen‐specific Tregs. Preliminary studies assessed the efficacy of CAR‐Tregs in preclinical models of autoimmune disease, GvHD, and inflammatory bowel disease. As an example and providing supporting evidence for the value of CAR‐Tregs as a therapeutic option for the treatment of colitis Blat et al. [71] demonstrated the efficacy of carcinoembryonic antigen (CEA) specific CAR Tregs in a CEA transgenic mice, using two disease models of colitis. In one model, CEA‐specific CAR Tregs were found to accumulate in the colon of the diseased mice suppressing the severity of colitis whilst in another model were able to decrease the subsequent colorectal tumor burden. These promising data have instigated the broader application of CAR Tregs in transplantation [72]. We and others designed a CAR‐Treg specific to donor HLA class I molecule (HLA‐A2), which is found universally throughout the graft. Supporting the findings of Noyan et al., we highlighted the superiority of CAR Tregs over polyclonally expanded Tregs at inhibiting alloimmune‐mediated damage of human skin xenografts [73]. Of note, adoptive transfer of similarly designed CAR‐Tregs specific to HLA‐A2 were able to prevent GvHD in studies of BMT [74]. These findings have stirred considerable interest in the use of a similar anti‐HLA‐A2 CAR‐Treg product in humans post kidney (Sangamo Therapeutics 2020) and liver transplantation (Quell Therapeutics 2021).

### Clinical application of Tregs in liver and solid organ transplantation: Clinical trials to date

Accumulating pre‐clinical data and clinical reports from immune monitoring studies in transplantation have attested to the value of Tregs in tolerance induction protocols. In this vein, low peripheral numbers of Tregs have been reported during acute rejection post‐liver transplantation [75] with Treg and FOXP3 mRNA enrichment noted in operationally tolerant allografts and peripheral blood [76, 77].

#### Treg cell therapy in liver transplantation

As we write there are several transplant centers around the world running clinical trials of Treg cell therapy in the setting of liver transplantation (**Table** **1** **)**. In 2016, Todo et al. conducted a study, aimed at tolerance induction in 10 adult living donor liver transplant recipients using a Treg‐based cell therapy [78]. Patients had a left lobe liver transplant together with a splenectomy. The immunosuppressive medications included: CNI, mycophenolate mofetil (MMF), and steroid on day 0, with a single dose of cyclophosphamide given on day 5, posttransplantation. Ex vivo generated donor‐specific autologous Tregs were produced by co‐culture of recipient lymphocytes with irradiated donor cells in the presence of costimulation blockade (anti‐CD80/86 antibodies). On day 13 posttransplantation, 0.23∼6.37 × 10^6^ cell/kg of the unpurified Treg‐enriched cell product was injected, after pre‐conditioning with cyclophosphamide. The immunosuppression withdrawal/discontinuation regimen included: discontinuation of MMF and steroids on month 1 after transplantation, subsequently, the CNI was tapered from month 6 to total withdrawal by month 18. The data presented showed the infused cells to be safe with no adverse events. Impressively, seven of 10 patients were completely weaned off immunosuppression without rejection. Of note, the three patients in whom withdrawal was not possible had a background of autoimmune liver diseases and developed mild rejection during immunosuppression withdrawal requiring maintenance on low‐dose immunotherapy

**Table 1:** Summary of clinical applications of Treg immunotherapy post‐liver transplantation

Investigators/Trial name/ID	Donor specific or polyclonal	Dose	Timing of infusion of Tregs	Status of Trial	Outcome
Todo and Okumura et al.[78] UMIN‐000015789.	Donor‐specific and co‐stimulation blockade	0.23‐6.37 × 10^6^ Tregs/ kg	Day 13 post transplantation	Completed	Safe. Immunosuppression withdrawal achieved in 7/10 patients. 3/10 had mild rejection episodes on withdrawal of immunosuppression
Sanchez‐Fueyo and Lombardi et al. [79] ThRIL NCT02166177	Polyclonal	0.5‐1 × 10^6^ Tregs/kg or 3–4.5 × 10^6^ Tregs/kg.	3/9 (enrolled at the time of transplantation) Treg infusion at 3 months post transplantation. 6/9 (enrolled at 6–12 months after transplantation) Treg infusion at 4 months after enrolment	Completed	Safe and well tolerated. 1 reported dose limiting toxicity (infusion reaction)
Feng, Bluestone, Tang and Kang. darTregs NCT02188719	Donor‐specific	50, 200, and 800 × 10^6^ Tregs/total dose	No patients received the product as the trial was terminated due to manufacturing issues	Terminated	No results available
Feng, Bluestone and Qizhi. ARTEMIS NCT02474199	Donor‐specific	400 × 10^6^ Tregs/total dose	2–6 years post transplantation	Completed	Results awaited
Feng LITTMUS‐UCSF NCT03654040	Donor‐specific	100‐500 × 10^6^ Tregs	Unknown	Recruiting	Results awaited
Markman LITTMUS‐MGH NCT03577431	Donor‐specific	2.5 to 125 × 10^⋀^6	Unknown	Recruiting	Results awaited
Wang et al. NCT01624077 1^st^ trial	Polyclonal	1 × 10^6^ Tregs/kg at intervals	Several intervals	Unknown	No results available
Wang et al. NCT01624077 2^nd^ Trial	Donor‐specific (MHC peptides)	1 × 10^6^ Tregs/kg at intervals	Patients recruited at 1–10 years post transplantation. Infusion of Tregs at several undefined time points	Unknown	No results available

At King's College London (KCL) we recently reported on ThRIL (NCT02166177), a randomized, open‐label, single ascending dose study of Treg immunotherapy in liver transplantation [79]. Trial participants were recruited pre‐transplantation or 6–12 months post‐transplant. Ex vivo autologous Tregs were isolated from peripheral blood and polyclonally expanded, using a 36‐day GMP‐compatible protocol in the presence of rapamycin [80]. This protocol developed by our group has previously shown the feasibility of the expansion of patient‐derived Tregs to clinically applicable numbers, whilst ensuring their stability and increased suppressor function. Seventeen patients consented pretransplantation and three injected with 0.5‐1 × 10^6^ Tregs/kg (having received anti‐thymocyte globulin (ATG) pre‐conditioning), while all six patients who consented at the later time point of 6–12 months posttransplantation received 3–4.5 × 10^6^ Tregs/kg. Of note, ATG was used as a “debulking” agent to achieve lymphocyte depletion, in line with preclinical models, already discussed, reporting that tolerance requires an effector T cell: Treg ratio in favor of Tregs [43]. The use of induction therapy in the setting of liver transplantation has increased over the past decade and has shown to be advantageous for candidates with renal dysfunction in delaying the introduction of CNIs, with known nephrotoxicity. Other additional consideration and in favor of the use ATG in Treg immunotherapy trials is based on studies showing the tolerogenic effects of ATG by promoting the thymic export of de novo Tregs [81] and expanding FOXP3+ T cells *in vivo*, whilst decreasing naïve T cells. Importantly, however, while ATG increases the pool of memory T cells, explaining the lack of overwhelming sepsis post such an induction therapy, this can pose a disadvantage as these cells are more resistant to Treg suppression compared to more naïve T cells [82] and can contribute to early graft injury. As such, patients were started on a CNI, tacrolimus, and steroids, with a strategy that included the introduction of an mTOR‐I at month two post ATG, and a month prior to the Treg injection, in order to maintain patients on a lower dose of CNI. Additional advantages of such a strategy are supported by the superiority of mTOR‐Is for the thymic export, survival, and function of Tregs as compared to CNIs [83]. In addition, other studies highlight that low‐dose mTOR‐I can potentiate the ability of Tregs to inhibit transplant arteriosclerosis development in a humanized mouse system [60]. The ThRIL study reinforced the safety profile of the injected Tregs with only a single reported infusion reaction, defined as dose‐limiting toxicity. Information gleaned from the immune monitoring data showed that post‐injection of 4.5 × 10^6^ Tregs/kg, the number of circulating Tregs was augmented with increased levels noted 1 month post‐infusion. A finding that was not seen when only 0.5‐1 × 10^6^ Tregs/kg were injected. It is noteworthy to mention that the longevity and migration of adoptive transfer of *ex vivo* expanded Tregs are not well understood. Data from mouse models of transplantation have shown the accumulation of Treg in the graft and draining lymph node [84, 85], with data specifically from murine models of islet transplantation reporting the detection of Tregs 14 days after infusion [86]. Moreover, in the setting of human Type I Diabetes and kidney transplantation the adoptive transfer of deuterium labeled, expanded Tregs saw a peak in circulating Tregs at 7–14 days after injection, with a rapid decrease thereafter. However, their persistence was noted at around 20% at 1 year after injection [87, 88]. Further immune monitoring data from ThRIL showed reduced anti‐donor T cell responses in the individuals who received 4.5 × 10^6^ Tregs/kg only, which remained suppressed at 4 weeks post Treg injection. Importantly, this display of donor‐specific T cell responses was similar to that observed by Todo et al. [78]. Data from preclinical models can further inform these findings, with the proposal that the donor‐specific hyporesponsiveness noted may be due to the selected persistence and/or expansion of Treg clones with anti‐donor alloreactivity, post‐Treg infusion [84]. This may also provide an explanation for the lack of pan‐immunosuppression that might have been predicted post‐infusion of polyclonally expanded Tregs.

Further clinical trials have seen the use of donor alloantigen reactive Tregs (darTregs) led by the UCSF group (NCT02188719) [89], generated by culture of FACS sorted CD4^+^CD25^+^ CD127lo Tregs with irradiated donor B cells at a ratio of 4:1, with polyclonal stimulation with antiCD3 antiCD28 beads on day 11 [57]. The clinical protocol, similar to that adopted in the ThRIL clinical trial, included the use of ATG as an induction agent, with the addition of an mTOR inhibitor, a strategy to minimize the CNI burden, prior to the infusion of autologous donor alloantigen‐specific Tregs at 3 months after transplantation. The trial intended to test deuterium‐labeled Tregs at 50, 200, and 800 × 10^6^cells/total dose, with this strategy of Treg labeling to allow the tracking of the infused cells. However, the trial was terminated due to manufacturing problems. The UCSF team is now conducting a subsequent trial, ARTEMIS (NCT02474199), with a different clinical design, including patients at 2–6 years after transplantation with stable liver graft. Approximately 400 × 10^6^ donor alloantigen reactive Tregs will be infused alongside immunosuppression withdrawal with the aim of achieving operational tolerance. The result of this trial is greatly anticipated.

#### Treg therapy in patients receiving other solid organ transplants

In addition to the optimistic results to date on the clinical application of Treg therapy in liver transplantation, it is important to also note the broader applicability of these cells in solid organ transplantation.

To date, numerous studies in the setting of kidney transplantation have contested the use of Tregs for tolerance induction strategies. One such study, the multicenter ONE study, not only assessed the safety and feasibility of polyclonal Treg cell therapy post living‐donor kidney transplantation, but also five other regulatory products, including regulatory macrophages and dendritic cells [90]. This trial included a reference group (RGT), where subjects received standard of care with the immunosuppressive regimen including basiliximab, tapered steroids, mycophenolate mofetil (MMF), and tacrolimus. In the group of patients who received the cell therapy product, basiliximab induction was substituted with the administration of the cell therapy product, with a plan for tapering of MMF. The primary endpoint was biopsy‐proven acute cellular rejection within 60 weeks after transplantation.

The data confirmed that while the incidence of acute cellular rejection was higher in the cell therapy arm at 16% versus 12% in the RGT group, there were fewer episodes of infections registered in the cell therapy trial. This was deemed to be secondary to the lower immunosuppression burden in this group as in the cell therapy arm patients were successfully weaned from MMG and maintained on tacrolimus monotherapy.

Although this trial was not designed or powered to compare the safety and efficacy of the various cell therapy products, it did support the safety of the injected cells and the potential that by adopting such strategies such as posttransplantation, this will allow the minimization of the immunosuppression at an earlier time point posttransplantation. The trial further highlighted that other than the use of Tregs for cell therapy application, other regulatory immune cells can be used for tolerance induction protocols.

As part of the ONE Study, several groups have now reported their experience on the feasibility, safety, and immune monitoring post Treg immunotherapy in the setting of kidney transplantation. The group from Berlin [91] specifically attempted to minimize immunosuppression post kidney transplantation. In their Phase I/IIa clinical trial, they tested three doses of Tregs at 0.5, 1.0, or 2.5‐3.0 × 10^6^ cell/kg with a clinical protocol designed to taper the triple immunosuppression to low dose tacrolimus until week 48. The trial further supported previous studies in reporting the feasibility of isolation and expansion of clinical‐grade autologous Tregs from kidney transplant recipients. From 40–50ml of blood the Tregs were polyclonally expanded over 23 days in the presence of rapamycin. Of note these cells were isolated using CD8^+^ T cell depletion and CD25^+^ T cell enrichment, using the CliniMACS Plus system. There was no report of dose‐limiting toxicity of the three Treg doses tested. The clinical results of the Treg treated cohort were compared to the ONE Study RGT. Both groups exhibited similar three‐year allograft survival (100%) and similar clinical and safety profiles. Of importance was the finding that in eight of the 11 patients (73%) monotherapy immunosuppression was achieved, while in the RGT group patients were on dual or triple immunosuppressive regimens.

In a subsequent study, our group, together with the team from Oxford University, reported on their Phase 1 clinical trial of Treg immunotherapy post kidney transplantation [92], conducted as part of the ONE Study. In this study 12 renal transplant recipients received the Treg cell product on day 5 post kidney transplantation. The trial dosed three patients at 1, 3, 6, or 10 × 10^6^ cells/kg with a minimum 2 weeks of observation for adverse reactions of dose‐related toxicity, prior dose escalation. Additionally, 19 patients received standard immunosuppression. In the latter group, immunosuppression was that of standard protocol, including induction with basiliximab and maintenance immunosuppression of tacrolimus, mycophenolate mofetil, and reducing doses of prednisolone. Of note, induction therapy was not included in the Treg therapy arm, with the rationale that this form of therapy would inhibit the infused Tregs [93]. The primary endpoints of the study were incidence of biopsy‐proven acute rejection and survival 60 weeks posttransplantation. The study objectives were to investigate the potential of *ex vivo* polyclonally expanded, cryopreserved autologous Tregs as a safe therapy in regulating the immune response, permitting the avoidance of induction therapy and immunosuppressive drugs. Treg immunotherapy was reported as being a safe strategy with acute rejection‐free survival at 100% in the Treg therapy arm as compared to the 78.9% in the control arm, 48 months after transplantation. Of note, Treg therapy allowed the minimization of the immunosuppressive burden with four patients successfully tolerating the withdrawal of mycophenolate mofetil and maintained on tacrolimus monotherapy. The data from the immunomonitoring aspect of the study revealed a 9–20‐fold increase in circulating Treg cell concentration in peripheral blood Tregs until 60 weeks (end of the observation period). Additionally, there was a reduction of overall proinflammatory monocyte numbers an increase in marginal zone B cells, an interesting finding in view of the reported regulatory function of these cells through the production of IL‐10 [94]. Of note, further deduction from the study was the identification of an immune phenotype of patients pretransplantation who were at high risk of unsuccessful *ex vivo* Treg expansion. This included a reduced frequency of lymphocytes at the start of expansion and a higher proportion of naïve Tregs. Additionally, the study highlighted the feasibility of successfully transporting a cryopreserved cell product between two clinical sites supporting the possibility of future multicenter trials.

Despite the many successes with the trials to date, Treg immunotherapy is currently an expensive therapeutic option, limiting its widespread availability. The future will not only see the optimization of the final product and process development but will require the collaborative efforts between academic institutions and industry to promote the widespread availability and broaden the applicability of the treatment in other disease settings. Additionally, the differences in the manufacturing process between the various Treg products have limited widespread comparison of the results in both the research and clinical arena. To date, there are nearly 30 ongoing clinical trials of Treg immunotherapy to treat inflammatory, autoimmune conditions and for tolerance induction [95], with a growing diversity in the protocols used, highlighting the need to develop standardized reporting systems, such as the published minimum information about Tregs (MITREG) to ensure consistency of the presented data, reproducibility and quality assurance [96].

Aside from the adoptive transfer of Tregs, other strategies may involve the *in vivo* modulation of these cells to either increase their number/ enhance their function *in vivo*. This is an attractive prospect, which may circumvent the expensive GMP‐ compatible manufacture of clinical‐grade Tregs.

### *In vivo* modulation of Tregs: Low‐dose IL‐2 for Treg expansion and tolerance

IL‐2 was originally discovered in the 1970s as a T cell growth factor, with subsequent work showing that mice lacking genes encoding for IL‐2, IL‐2Rα, or IL‐2Rβ had a paucity of Tregs and developed lethal autoimmunity [97, 98]. It is now well recognized that IL‐2 is a critical factor for Tregs and required for their function and stability [99]. Moreover, focus on cellular metabolic pathways [100, 101, 102] has revealed a crucial role that IL‐2 plays with regards to its influence on Treg metabolic functioning (reviewed in [103].)

Administration of subcutaneous low dose IL‐2 has yielded promising results in the treatment of autoimmune conditions such as chronic refractory GVHD, HCV–induced vasculitis, and type 1 diabetes [104, 105, 106]. However, the short half‐life of IL‐2 [98] and its off‐target effects limit its efficacy. In this regard, it is important to note that while IL‐2 predominantly activates cells expressing high‐affinity receptors, (IL‐2R [CD25], IL‐2R [CD122], and γc [CD132]); it can also activate memory CD8^+^ cells and natural killer cells with low‐affinity IL‐2 receptors [107, 108].

To circumvent these issues *Boyman et al*. [109] devised a method that included the combination of IL‐2 with JES6‐1, an anti‐IL‐2 monoclonal antibody (mAb), previously shown to result in the increased half‐life of IL‐2 with the selective expansion of Tregs with little or no effect on other cells [110].

The first studies to use this strategy in transplantation were performed in experimental models of pancreatic islet cell allografts [111], with reports that increased Treg numbers could be achieved in the spleen on day 3 after three daily intraperitoneal administration of IL‐2 (1 mcg) in combination with 5 mcg of JES6‐1 (IL‐2 mAb). The authors further showed that pre‐treatment with anti‐IL‐2 complex (IL‐2c) with this regimen resulted in the acceptance of the fully MHC‐incompatible pancreatic islet cells.

Additionally, our group has recently demonstrated the advantage of IL‐2c in combination with Treg adoptive transfer, reporting that such a strategy resulted in the preferential expansion of adoptively transferred donor‐specific Tregs [84]. This resulted in a synergistic effect, which extended graft survival in a skin transplant model with a single MHC mismatch. Furthermore, other reports have investigated the combination of low dose IL‐2 therapy with interventions that block effector T cell responses, so as to provide another strategy to prevent the off‐target effects of this therapy.

In line with this, *Pilon et al*. investigated the role of low‐dose IL‐2 in preventing skin allograft rejection. In support of a combination therapy, they showed that co‐administration of IL‐2 and rapamycin promoted skin graft survival, as compared to IL‐2 treatment alone, with the combination therapy proving superior by inhibiting the expansion and activation of conventional CD4^+^ cells [112]. Moreover, using this strategy, Pilat et al. have recently shown that combining low doses of IL‐2 complex with rapamycin and anti‐IL‐6 mAb resulted in the long‐term survival of MHC‐mismatched skin allografts [113].

In accordance with these studies, we have recently investigated the effects of combination therapy with low dose IL‐2 and CNIs. CNIs are known to effectively control T effector cell responses, although at the cost of reducing the Treg pool, with an as yet undefined mechanism. Our group has recently shown that CNIs selectively promoted the apoptosis of resting and activated Treg subsets [114]. In this study, we proposed that these effects were caused by reduced availability of IL‐2, rather than disruption of the NFAT pathway, secondary to CNI use. In support of this, we showed that Tregs remained capable of translocating NFAT, despite high CNI levels, with exogenous IL‐2 promoting their growth and survival via anti‐apoptotic Bcl‐2 expression.

Furthermore, using a preclinical model of skin transplantation, we showed the increased allograft survival with the addition of IL‐2 to CNI treatment, and reported the accumulation of Tregs within the graft. As such, supplementary IL‐2 therapy with CNIs puts forward a promising strategy that not only allows for the effective control of cytopathic T cell responses, but also enhances the pool of suppressive Treg subsets.

In 2016 at KCH we embarked on the ‘LITE’ (NCT02949492), an open‐label prospective single arm clinical trial, with the aim of investigating the effects of low dose IL‐2 treatment in liver transplant recipients, receiving CNIs. This trial is set to investigate whether this combination therapy will allow for the complete discontinuation of immunosuppression, with the results much anticipated.

The studies highlighted to date support low‐dose IL‐2 and/or combination therapies as an optimal immunomodulatory approach for the prevention of transplant rejection. Additionally, they inform us of the many questions that still need to be answered, such as how best to tackle the off‐target effects, the best combination therapy, and the dose of IL‐2 used.

### Use of Tregs beyond transplantation: Treg‐based therapy in liver diseases

Aside from their use in the setting of transplantation, the future will see an in‐depth understanding of Treg biology in patients with liver diseases of differing aetiologies, with the intention of devising novel therapies to halt/ prevent liver disease progression. In this regards, studies have shown variations in Treg numbers in patients with liver diseases, with a reduction in Treg frequency reported in patients with alcoholic hepatitis [115] and a decreased ratio of Tregs:Th17 cells in patients who progress from non‐alcoholic fatty liver disease (NAFLD) to non‐alcoholic steatohepatitis (NASH) [116]. Moreover, there has been a wealth of data proposing a numerical and function defect in Tregs in patients with autoimmune hepatitis (reviewed in [117]) with the rationale of using Treg therapy, either through the *in vivo* modulation or their adoptive transfer, to tip the balance in favor of immune regulation over autoreactive effector responses,

In line with this, a recent clinical trial, showed the feasibility of Treg isolation from leukapheresis products from patients with AIH [118]. In this study*, Oo et al*. used ^111^indium tropolonate labeling of Tregs and showed their homing and retention at the liver site for up to 72 h. The study, however, was not powered to demonstrate efficacy, with the maximum dose of injected cells being 86 × 10^6^. Previous studies in the setting of diabetes have used a total of up to 256 × 10^6^ Tregs [87], highlighting that future trials in the setting of AIH will require expansion of the isolated cells prior their infusion to optimize efficacy.

Additionally, low dose IL‐2 therapy has been used as an alternative strategy to modulate *in vivo* Tregs. In a prospective, open label phase I‐IIa study of 46 patients with a variety of autoimmune diseases [119] including patients with AIH and sclerosing cholangitis, a dose of 1million IU/day of IL‐2 was given for 5 days, followed by fortnightly injections for 6 months. Immunomonitoring data reported specific Treg expansion and activation in all patients tested and across all disease categories, supporting the use of low dose IL‐2 in various autoimmune and inflammatory diseases.

More specifically, we have reported our findings on the treatment of two patients with refractory AIH with low dose IL‐2 [120]. The first patient was a 21‐year‐old woman with type 1AIH, diagnosed at the age of 13 and despite triple therapy, had required repeated intravenous steroids for AIH flares. The second patient was a 57‐year‐old woman with type 1 AIH diagnosed at the age of 52 and despite treatment with steroids and MMF, and multiple flares whilst n this management, refused initiating tacrolimus due to the long‐term toxicity concerns. Both patients had persistent disease activity prior the treatment, as assessed by serum biochemical and liver biopsy. A dose of 1millionIU/day s/c was given for 5 days, followed by three weeks washout, with both patients receiving 6 cycles.

The immunomonitoring revealed an increase in the proportion of circulating Tregs in both patients by day 4 after each cycle of low dose Il‐2, peaking at day 9 and returning to baseline values at day 28. Additionally, this strategy appeared to increase the IL‐2 sensitivity of Tregs and corrected the suboptimal phosphorylated STAT5 response, described in AIH [121]. Of importance, the effects of low dose Il‐2 were noted to be on the Treg population as compared to the non‐Treg immune compartment, which is encouraging in view of potential concerns with the off‐target effects of this therapy. The clinical evaluation of the patients revealed that unlike patient 2, where a decrease in AST and IgG levels to within a normal range were noted, there was lack of response to this treatment strategy by patient 1. The lack of response in patient 1 was deemed to be secondary to the cirrhosis, with animal data supporting that intrahepatic Treg expansion is compromised in the presence of severe liver damage [122]. This poses further questions with regards the clinical application of such a therapy such as the timing of therapy during the disease course, and frequency and dose of therapy.

## Concluding remarks

The mortality and morbidity associated with chronic immunosuppression have been the motivation behind drug withdrawal trials and tolerance induction strategies. In this review we have highlighted the phenomenon of operational tolerance following liver transplantation, summarizing the outcome of immunosuppression withdrawal trials and strategies to accelerate tolerance induction, largely through Treg adoptive cell therapy. Results from clinical trials to date have shown that the adoptive transfer of such regulatory cells may have significant potential in promoting tolerance induction and recent optimization strategies including CAR technology will prove to further enhance the efficiency and efficacy of Treg tolerance protocols. While research in the Treg field continues to advance future optimization of Treg‐based immunotherapy, rests on the further characterization and understanding patient‐derived Tregs. The information gleaned from understanding of the Treg homeostasis, proliferative capacity, stability, migration, and function within the inflammatory microenvironment and/ or in the presence of immunosuppression in patients posttransplantation, will further direct future immunotherapy trials. As eluded to earlier, the last decade has seen advancement in the field of immune metabolism with studies on the various metabolic pathways used by Tregs for their proliferation, suppressor function [102], and migration [123] (further reviewed in [124]). Additionally, much research is now focused on the behavior of these cells within a microenvironment deficient in certain metabolites that is necessary for their *in vivo* function. A more complete understanding of Treg metabolism in health and disease will allow for the targeted modulation of Treg metabolic pathways *in vivo*. This may pose an attractive future strategy for the improvement of the adoptively transfused cells or the use of combination therapies. The next few years will see focused efforts on monitoring their function, migration, stability, and survival following injection so as to further optimize the quality of these cells and continue to enhance their design.

## Conflict of interest

The authors declare no commercial or financial conflict of interest.

**Abbreviations**
APC: antigen‐presenting cells
ATG: anti‐thymocyte globulin
BMT: bone marrow transplantation
CNI: Calcineurin inhibitors
CAR: chimeric antigen receptors
CAAR: chimeric autoantibody receptor
CEA: carcinoembryonic antigen
DC: dendritic cell
FACS: fluorescence‐activated cell sorting
FOXP3: Forkhead box protein 3
GMP: good manufacturing practice
GVHD: graft versus host disease
HLA: human leucocyte antigen
LSEC: liver sinusoidal endothelial cells
MBP: myelin‐basic protein
MMF: mycophenolate mofetil
mTOR: mechanistic target of rapamycin inhibitor
TCR: T cell receptor
TSDR: Treg specific demethylated region

## Data Availability

The data that support the findings of this study are available from the corresponding author upon reasonable request.
